# Microstructure and properties analysis of the brazing alloy prepared from recycled E-waste

**DOI:** 10.3389/fchem.2022.1038555

**Published:** 2022-10-05

**Authors:** Jiao Yang, Li Bao, Weimin Long, Sujuan Zhong, Jian Qin, Ruilin Qiao

**Affiliations:** ^1^ State Key Laboratory of Advanced Brazing Filler Metals and Technology of Zhengzhou Research Institute of Mechanical Engineering Co., Ltd., Zhengzhou, Henan, China; ^2^ Beijing University of Science and Technology, Beijing, China

**Keywords:** electronic waste alloy, copper based brazing filler metals, microstructure, wettability, mechanical properties

## Abstract

In order to realize the efficient and comprehensive utilization of e-waste resources and short process preparation of alloy brazing materials, this study has analyzed the microstructure and properties of e-waste recycled brazing alloys by the analysis methods of inductively coupled plasma emission spectrometer, differential scanning calorimeter, scanning electron microscope, metalloscope, X-ray diffractometer, micro-hardness tester. Experimental results showed that phase compositions are significant differences between the alloys prepared by the recycled e-waste and the pure metals. The circuit board recycling alloy mainly consisted of α-Fe dendrites, (Cu, Sn) phases, Sn-rich phases and Cu matrix, while the alloy obtained by pure metals is composed of (Cu, Sn) phase, Sn-rich phase and Cu matrix. The melting temperature of alloy obtained by melting the circuit board is in the range of 985.3°C–1,053.0°C, which was wider and higher than that of alloy obtained by pure metal smelting. The shear strengths of the joints brazed by the brazing alloys prepared by the recycle e-waste and pure metals are 182.21 MPa and 277.02 MPa, respectively. There is little difference in hardness between the two types of brazed joints. In addition, there are a large number of precipitated phases in alloy obtained by the recycled circuit board, owing to the precipitation strengthening mechanism. The main strengthening mechanism of alloy obtained by pure metals is solid-solution strengthening. The paper focused primarily on alloy obtained by melting the circuit board and studying the specific composition, melting temperature, structure, and properties of alloys formed by melting the circuit board and pure metals. Meawhile, the size, morphology and other microstructure evolution of the second phase of brazing alloy were investigated to provide theoretical guidance for the brazing alloy in the subsequent actual production process.

## Introduction

Electronic waste, also called e-waste, includes a variety of obsolete computers, waste air conditioning, waste washing machines, and other household appliances, as well as some communication equipment. China generates a vast quantity of e-waste, which is continually increasing (Guo and Yan, 2017; Kang et al., 2020; Rao et al., 2020). It is rich in various metals, non-metallic substances, and organic materials in waste electrical and electronic equipment. With the global shortage of resources, e-waste as a critical “urban mine” attracts much attention and concern from society. E-waste is mainly composed of plastics, inert oxides, and metals. Taking circuit boards as an example, the metal content is high in circuit boards, with the majority of being common metals like Cu, Sn, Fe, Ni, Al, and Zn. Mo, Sb, and rare and precious metals, such as, Au and Ag (Jiang et al., 2016), have a comparatively low concentration. The research reports that a number of methods of recovering metal from circuit boards in China and abroad are dominated by physical separation extractive techniques such as magnetic separation, electrical separation, eddy current separation (Zheng et al., 2016; Gu et al., 2019), pyrometallurgy (Park and Kim, 2019; Habibi et al., 2020; Yue et al., 2021), hydrometallurgy (Díaz-Martínez et al., 2019), Bio-hydrometallurgy (Gu, 2016), and other extractive techniques. Due to the characteristics of e-waste, including its large quantity, complicated constituents, great harm, high potential value, and difficulty in treatment (Cao et al., 2003; Zhu et al., 2018), the recovery of valuable metals is the focus of attention for metal resource recovery from e-waste. Additionally, most of them are mainly recycling valued metals, which have many problems, such as long process flows, high energy consumption, and the generation of new pollutants like electrolytes and residue.

There is a high demand for multi-component alloys in China. For example, China consumes around 100,000 tons of copper-base brazing filler metals annually. The application of multi-component alloys is very wide. It is primarily employed in the production of diamond tools. Specifically, it could be widely applied in numerous engineering technical fields, such as automobiles, engineering machinery, aeronautics, and astronautics. Multi-component alloys usually contain Cu, Fe, Sn, Ni, Ag, Zn, and other elements. Consequently, the highest content of Cu in circuit board alloys can be used as the main component of brazing materials to produce copper-based brazing alloys. The brazing properties of alloys can be increased by adding Zn, Ni, and Sn elements (Long et al., 2017; Rene et al., 2021; Yang et al., 2022). Long Weimin (Long et al., 2017) studied the copper-base brazing alloys containing silver to braze. It was concluded that the addition of silver could improve the wettability of copper-base brazing alloys, hence greatly reducing the thickness of the copper-phosphorus/carbon steel interface compound layer and efficiently enhancing the strength and toughness of the brazed joint. Yang Jiao and Bao Li (Yang et al., 2022) summarized several typical copper-based brazing filler metals and mentioned that Zn additive can improve the wettability of copper-based brazing alloy and reduce the melting temperature of alloy. Ni element can refine crystalline, increase sintered body density, strengthen phase microstructure, and prevent crack propagation.

Most of the metals in circuit boards are feasible as components of the multi-element alloy brazing alloy. Besides, this may lower the melting point, solve problems of uneven composition, poor alloying extent and poor stability (Long et al., 2015). If the e-waste recycling target is used as an alloy preparation, the difficulties associated with refining individual metals can be avoided one by one. So that most of the metal elements can be recycled and utilized to achieve efficient recycling and comprehensive utilization of resources. However, most researches have concentrated on how to extract the required singlet metals from electronic wastes, but few reports on the composition, microstructure, and properties of alloy obtained by melting the circuit board. Therefore, this paper has focused primarily on melting the circuit board and studying the specific composition, melting temperature, structure, and properties of alloys formed by melting the circuit board and pure metals (Xue, 2018), (Wang et al., 2021). Meawhile, the size, morphology and other microstructure evolution of the second phase of brazing alloy were investigated to provide theoretical guidance for the brazing alloy in the subsequent actual production process.

## Experiments

### Materials

Circuit boards from discarded mobile phones served as the basic material. The recycled alloy is obtained by shearing, crushing, pretreatment, and high-temperature smelting. In addition, based on the composition ratio of brazing alloy obtained by melting the circuit board, materials including copper plate and tin block with a purity of 99.99%, Cu-37% Zn and Fe-36% Ni master alloys were utilized to quantitatively melt the alloy using induction heating equipment. The resulting alloy was then compared to that obtained by melting the circuit board.

### Methods

After the melting process was completed, the alloy elements were quantitatively analyzed by an inductively coupled plasma emission spectrometer (ICP-OES) to measure the types and contents of the contained elements. The melting points of the two alloys were measured by differential scanning calorimeter (DSC), and the specimens were taken at 30 mg, heated to 1,300°C at a heating rate of 15°C/min; Then, the solid and liquid phase lines were determined based on the DSC curves; The metallographic specimens and hardness specimens were intercepted by wire cutting, inlaid, polished, and then observed by a JEOL JSM-700F scanning electron microscope (SEM). Polycrystal X - Ray Diffractometer was used for detection. The target used in the experiment was copper target (Cu-Kα Rays, λ= 0.15405 nm), the current and voltage were 40 mA and 40 kV respectively, and the scanning angle ranged from 30° to 90°. It combined with energy spectrum analyzer (EDS)microstructure and phase of the alloy were analyzed; HV-1000 microhardness tester was used to measure the specimen’s Vickers hardness; Loading load was 0.1 kN, the loading time was 10 s, and a point was selected at every 0.2 mm interval, with 10 points being the average value of the hardness test.

According to the national standard GB/T 11364-2008 “brazing material wettability test method,” the Wetting test was carried out on the molten brazing alloy. The brazing alloys were wholly melted, maintained for 10 s, and then air-cooled. After wetting and spreading, the spread area of the brazing alloy was measured by image analysis software. The test was repeated five times, with the results being averaged. After welding the two brazing alloys onto stainless steel, joint shear tests were carried out. The shear strength of the joint specimen was evaluated using a universal testing equipment. The shear test was repeated three times, and the results were averaged.

## Results and discussion

### Composition analysis

The main chemical composition of brazing alloy was obtained by melting the circuit board sample No. 1. According to the composition ratio of the melting alloy, melting, the main chemical composition of the alloy No. 2 sample is shown in Table 1.

**TABLE 1:** Main chemical compositions of the two alloy specimens (wt%).

Element	Cu	Sn	Fe	Ni	Pb	Ag
Sample 1	Bal.	4.85	3.29	0.66	0.82	0.3
Sample 2	Bal.	4.48	0.53	0.72	31ppm	0.13

Compared the composition of alloy obtained by melting the circuit board and pure metal, it was found that the alloy obtained by pure metal iron content has a quit low iron content. In contrast, the proportional melting alloy added to the iron content of 3.5% results in measured iron content of 0.53% at the end of melting. According to the Cu-Fe binary phase diagrams, it can be seen that the solubility of Fe in Cu is low at room temperature, and the melting process is heated by conventional methods and cooled by air-cooling, resulting in insufficient iron content.

### DSC analysis

**FIGURE 1:**
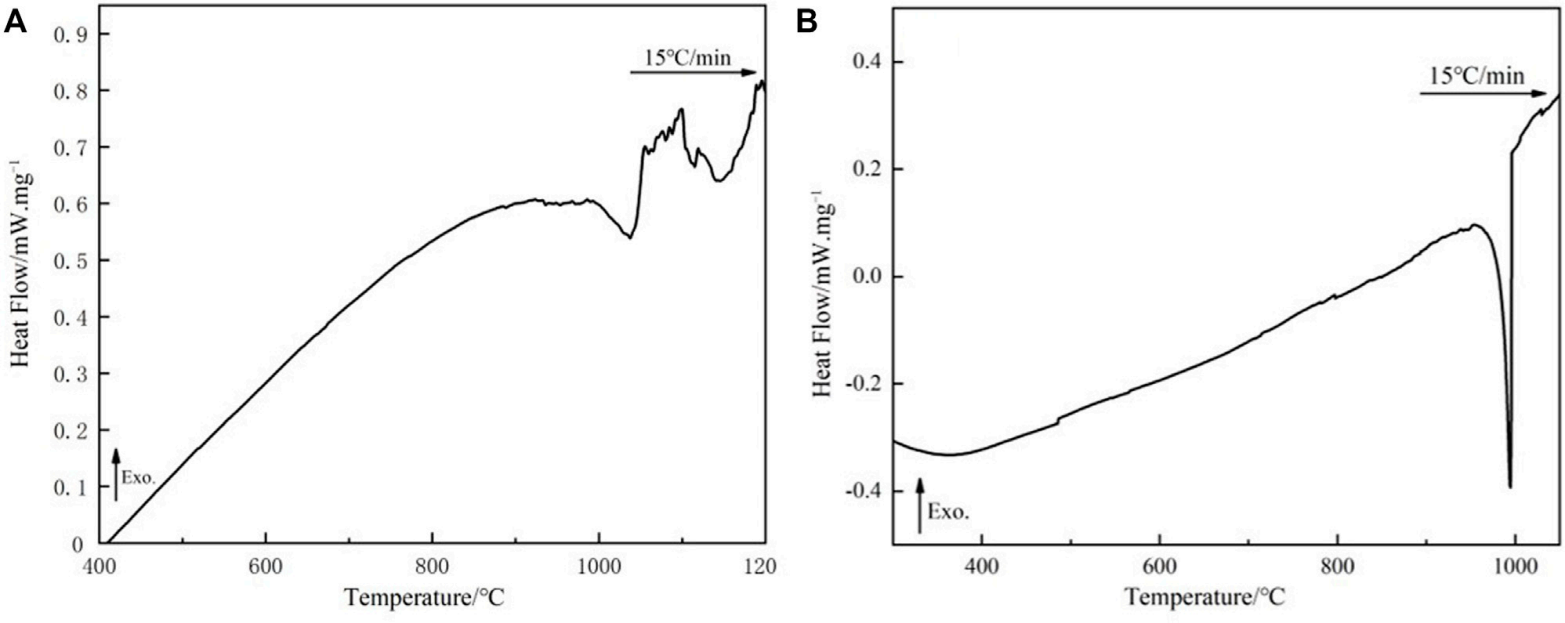
DSC curves of brazing alloys: **(A)** melting circuit board and **(B)** melting pure metals.

**FIGURE 2:**
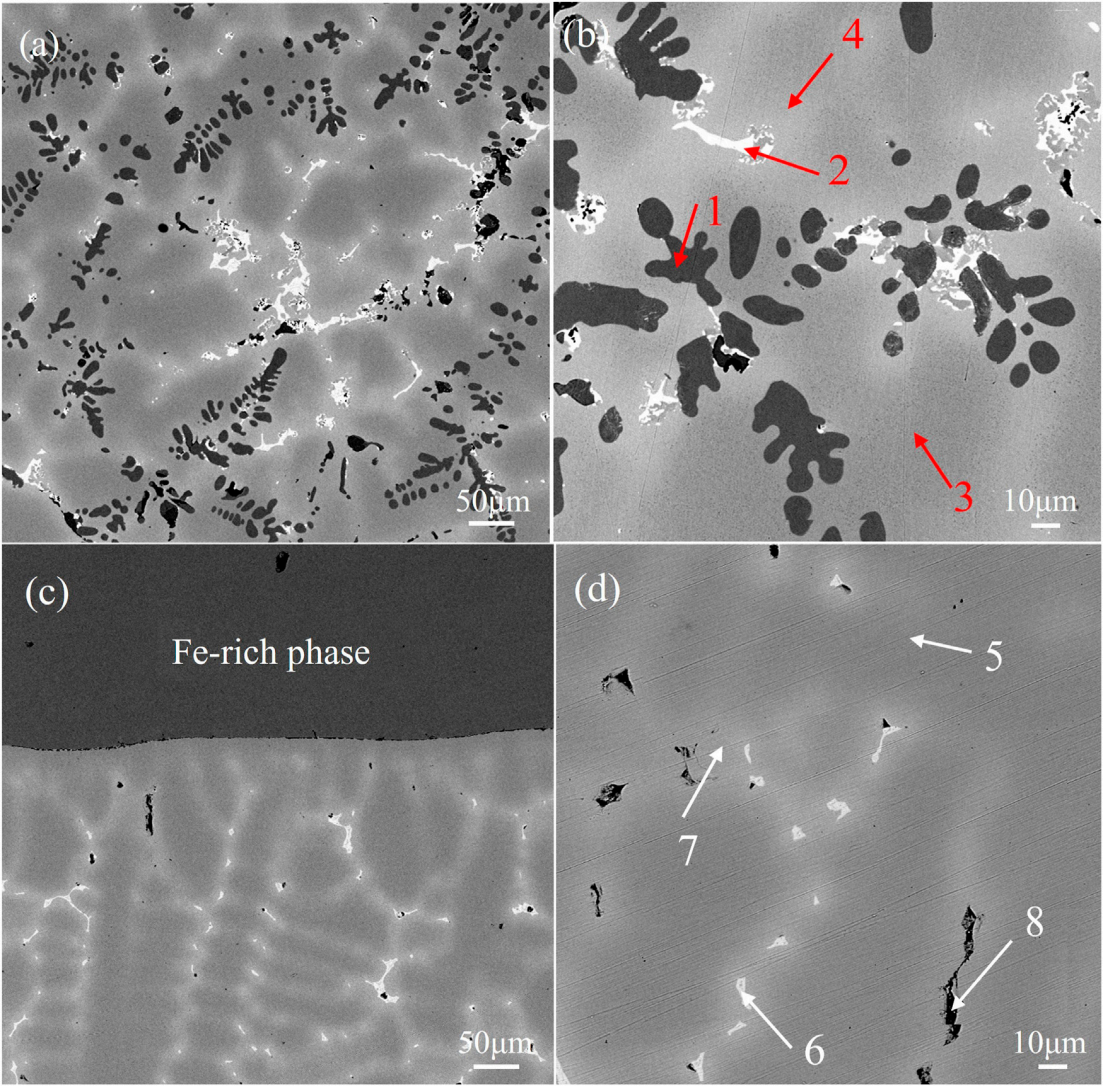
Microstructure of alloys **(A) (B)** alloy obtained by melting the circuit board and**(C) (D)** alloy obtained by pure metals.

Figure 1 depicts the melting curve for the alloy obtained by melting the circuit board, which was analyzed by using DSC analysis with alloy obtained by the pure metal. Figure 2B reveals alloy obtained by the pure metal has only one endothermic peak, with a peak temperature of 994.5°C. Correspondingly, it has a narrow melting temperature range of 941.0–996.1°C and good wettability. As shown in Figure 2A, the alloy obtained by the circuit board has two reaction heat endothermic peaks, with peaks at 1,037.2 °C and 1,144.7°C, corresponding to the heat endothermic peak temperature of 985.3–1,053.0°C and 1,099.4–1,189.2°C, respectively. The difference between this temperature melting interval and the melting temperature interval in the phase diagram is obvious. This difference in the melting temperature interval is related to experimental conditions and the change in elemental composition. The combined alloys obtained by the pure metal have an exothermic peak, showing that only one reaction occurs; While the.

Recycling alloy has two exothermic peaks, indicating that two reactions occur. Compared to the melting characteristics of alloy obtained by the pure metal, it indicates that the second peak of the recycling alloy might be a non-metallic reaction occurs. Therefore, the melting temperature range of the alloy obtained by melting the circuit board should be 985.3–1,053.0°C, which is higher than the alloy obtained by the pure metal.

### Microstructure analysis of brazing alloys

**FIGURE 3:**
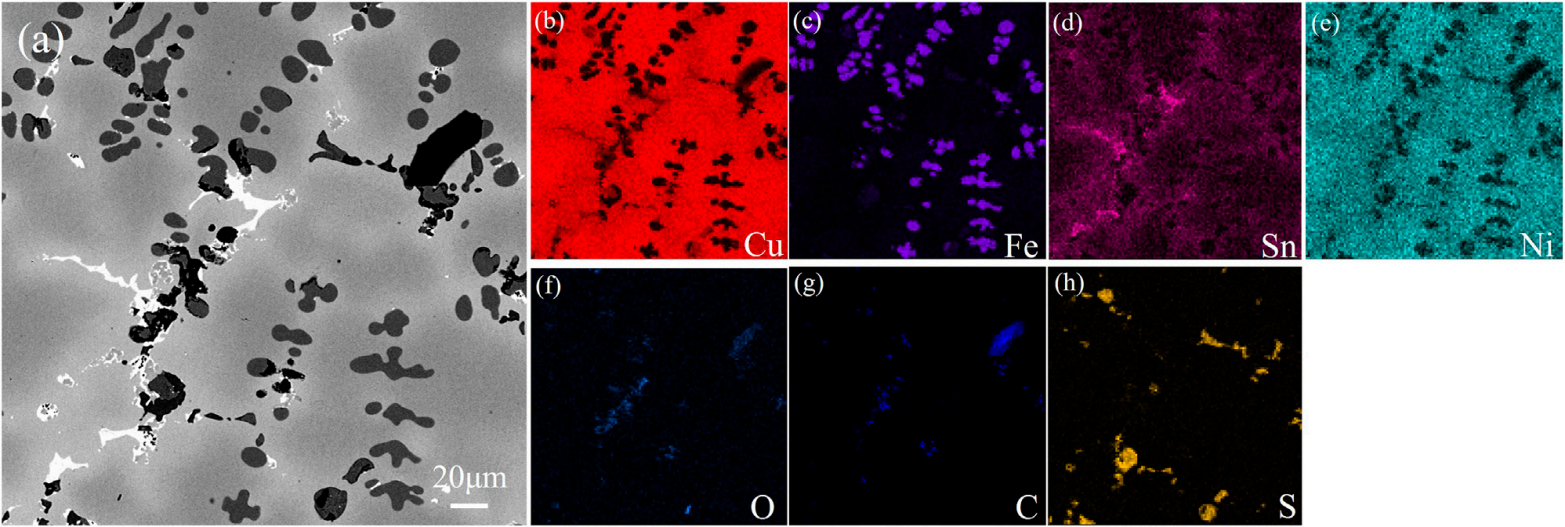
Microstructure and EDS distribution of alloy obtained by melting the circuit board.

Figure 2 shows the microscopic morphological SEM images of the two brazing alloys. Figure 2A is a low magnification image of alloy obtained by melting the circuit board, which shows the distribution of black dendrites, bright white phase, peripheral gray-white phase, and dark gray Cu matrix in the figure. The dendrites are precipitated on the Cu matrix with a volume fraction as high as 79.12%, showing obvious dendrite segregation. This resembles the casting microstructure image of Cu-Fe alloy observed by Yue Shipeng et al. (Xue, 2018), displaying the characteristics of uneven distribution; Figure 2B shows the magnified image of Figure 3A, and the marked points 1–4 use the EDS elemental analysis. The analysis results are shown in Table 2; The black phase is the Fe-rich intermetallic compounds, and the bright white phase is the Sn-rich phase combined with the relevant references and EDS analysis. Figure 2C is a low-power electron microscope image of an alloy sample obtained by melting the pure metal. Large pieces of unmelted Fe-Ni alloy can be seen to occur a severe macroscopic segregation which is easy to produce when melting Cu alloy with high Fe content. Magnification of the copper matrix can be seen in the high magnification image, as shown in Figure 2D; It can be seen that alloy obtained by melting the.

**TABLE 2:** Analysis results of energy spectrum components at each point (wt%).

Region	Cu	Fe	Ni	Sn	Ag	Pb	Zn	C	O
1	15.64	79.12	3.65	0.61	—	0.43	0.19	0.36	—
2	57.02	1.08	4.12	30.54	0.97	0.41	—	0.50	5.35
3	90.28	2.17	1.17	3.26	0.24	0.72	—	0.97	1.24
4	80.38	1.04	1.28	11.76	0.41	0.59	—	0.69	3.85
5	93.50	1.61	1.58	1.72	0.23	—	0.35	1.01	—
6	59.77	0.17	1.47	30.04	1.78	—	1.03	0.77	4.97
7	81.49	1.07	12.76	6.56	0.89	—	0.40	0.98	2.21
8	73.54	0.41	—	12.68	0.91	—	—	3.82	8.63
9	3.41	62.77	31.48	—	—	—	—	0.75	1.59

Pure metal has the same the bright white phase, peripheral gray-white phase and dark gray Cu matrix composition, except for the lack of black phase in the microstructure of the alloy obtained by melting circuit boards. The composition of points 5-8 and 1-4 in Table 2 shows that the bright white phase is the Sn-rich phase. However, compared to the thinner and more uniform alloy sample size obtained by melting the circuit board, the Cu matrix appears dark gray. There is no Fe-rich phase precipitation, and the solid solution degree is better.

**FIGURE 4:**
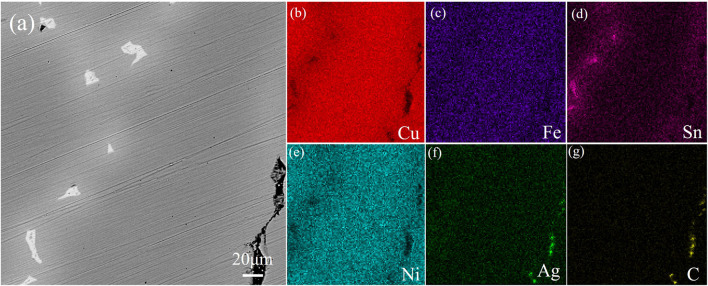
Microstructure and EDS distribution of alloy obtained by pure metals.

**FIGURE 5:**
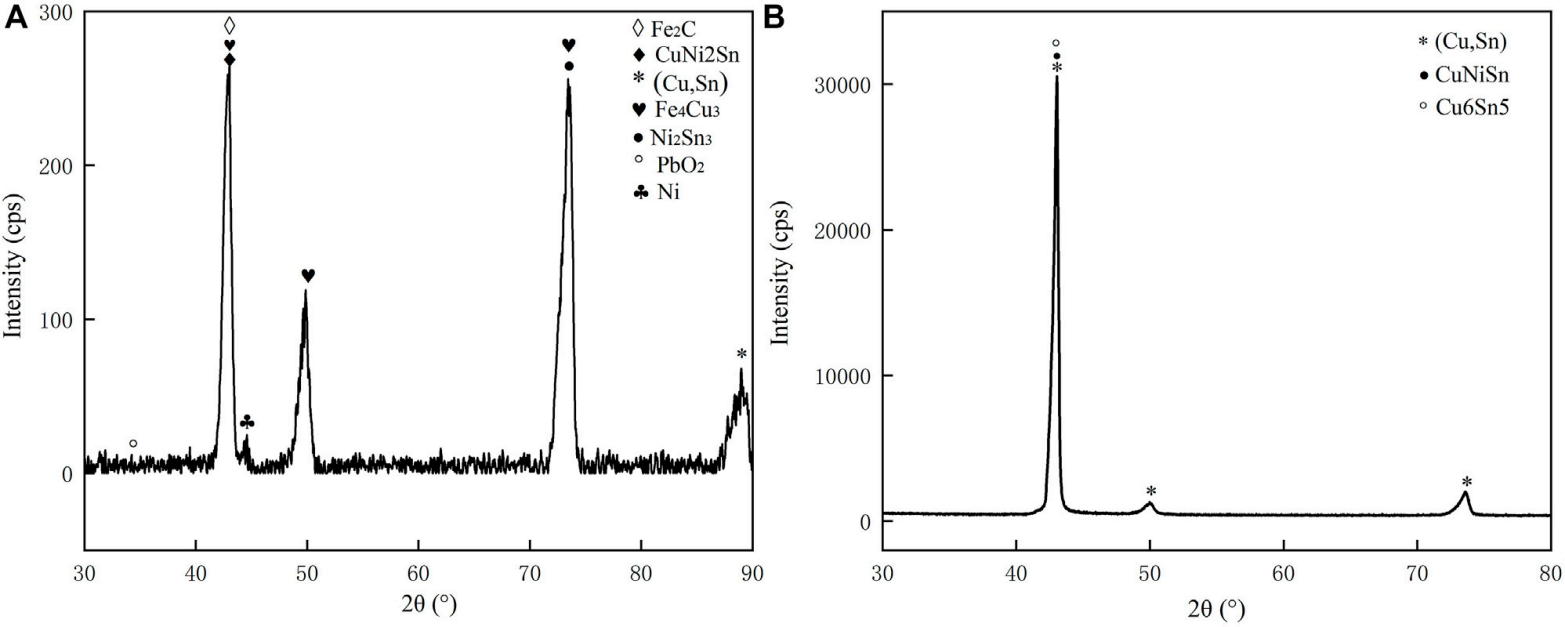
XRD analysis **(A)** alloy obtained by melting the circuit board **(B)** alloy obtained by pure metals.

Figures 3 and 4 show the photos of area scan by SEM and EDS about the two alloy samples. The photos of area scan by SEM in Figure 3A and Figure 4A. The main element of the black precipitated phase in Figure 3C mainly contains Fe. Moreover, based on the XRD patterns in Figure 5 and some references, the precipitated phase should be α-Fe dendrites, and Ni elements in Figure 3E are less solidly soluble in α-Fe dendrites; More Ni elements are solidly soluble in the dark gray Cu matrix in Figure 3B with a small amount of Sn in Figure 3D, which is uniformly diffused; Most of the Sn elements form a bright white (Cu, Sn) phase, in which O elements in Figure 3F are also distributed, forming compound inclusions; The surrounding diffuse distribution of Sn-rich phase, with a medium Sn content; Non-metallic impurities such as C and S can be seen in Figure 3G,H , which may be residues from the incomplete separation of e-waste or inclusions generated during the pyrometallurgical melting process. As can be seen from Figure 4, microelement Fe in Figure 4C is diffusely distributed on the Cu matrix in Figure 3B, and most Ni elements in Figure 3E are solidly dissolved in the Cu matrix; The precipitated phase is only the bright white phase formed by the enrichment of Sn elements, while Ag in Figure 3F, C in Figure 3G elements are gathered and distributed on the black irregular shape. The appearance of C may be from the inclusion produced in the smelting process.

### XRD analysis

The XRD patterns of the alloys with varied compositions are shown in Figure 5. As can be seen, the content of precipitated iron leads to some differences in the diffraction patterns of the two groups of alloys, indicating a shift in their phase composition changes, with the higher content of Fe precipitated in the form of α-Fe dendrites. In contrast, the lower Fe content primarily exists as a solid solution in the copper matrix, which is consistent with the previous EDS analysis. In addition, it is evident from its diffraction pattern that the alloy obtained by melting the circuit board consists of α-Fe dendrites, (Cu, Sn) phase, Sn-rich phase, and Cu matrix. Alloys obtained by the pure metal consist of (Cu, Sn) phase, Sn-rich phase, and Cu matrix, which is different with the phase composition of recycled samples.

### Hardness analysis

As demonstrated in Table 3, alloys obtained by recycling and melting the circuit board were subjected to hardness analysis with alloys obtained by the pure metal. The average microhardness of the black phase in the recycling alloy for circuit boards is 159.30 HV_0.1_, which is relatively high. The microhardness at the copper matrix of the two brazing alloys is 112.68 HV_0.1_ and 104.83 HV_0.1_, respectively. Overall, the recycling alloy for circuit boards has a little higher than the.

**TABLE 3:** Microhardness at each area of alloys obtained by melting the circuit board sample 1 and pure metals sample 2.

Sample	Location	1	2	3	4	5	6	7	8	9	10	Average/HV10
Sample 1	Matrix	99.8	111.7	109.6	121.4	105.9	102.8	106.3	112.6	116.5	115.3	112.68
	Black phase	163.2	164.6	155.8	138.6	153.5	177.8	145.0	134.7	183.9	133.0	159.30
Sample 2	——	98.3	96.8	117.5	95.5	95.9	109.6	105.9	120.0	108.4	100.2	104.83

Melting alloy for pure metal. According to the analysis depicted in Figure 3, the high hardness of alloys obtained by recycling and melting the circuit board is a result of the α-Fe dendrites created when the Fe content is excessive. The precipitation of Fe significantly increases the hardness of the alloy, but the overall hardness does not change significantly.

### Wettability

**FIGURE 6:**
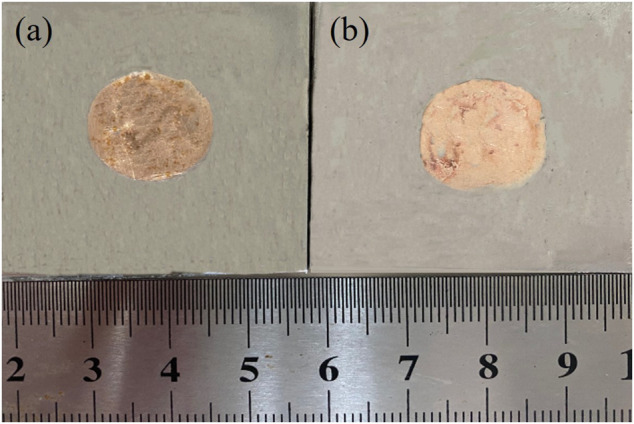
Spreading area of two alloys on 304 stainless steels: alloy obtained by melting the circuit board **(A)** and pure metals **(B)**.

Two brazing alloys were used for wetting test in stainless steel at a temperature of 1,080°C, and Figure 6 depicts the macroscopic morphology after the wetting experiment. Figure 6A is the spreading area of alloys obtained by melting the circuit board, with an average of 198 mm^2^, while Figure 6B is the spreading area of brazing alloy obtained by the pure metal, with an average of 180 mm^2^. The alloys obtained by melting the circuit board than alloy by melting pure metal in wettability is slightly better. Nevertheless, based on the DSC curve, the melting temperature interval of alloy obtained by the pure metal is narrower. On the one hand, it could be due to the higher temperature, on the other hand, the presence of the Fe element enhances the wettability of the brazing alloy on 304 stainless steels. The strong activite effect of brazing flux played a good role in de-filming, protecting the liquid brazing alloy, and reducing interfacial tension.

### Properties and microstructure of brazed joint

**FIGURE 7:**
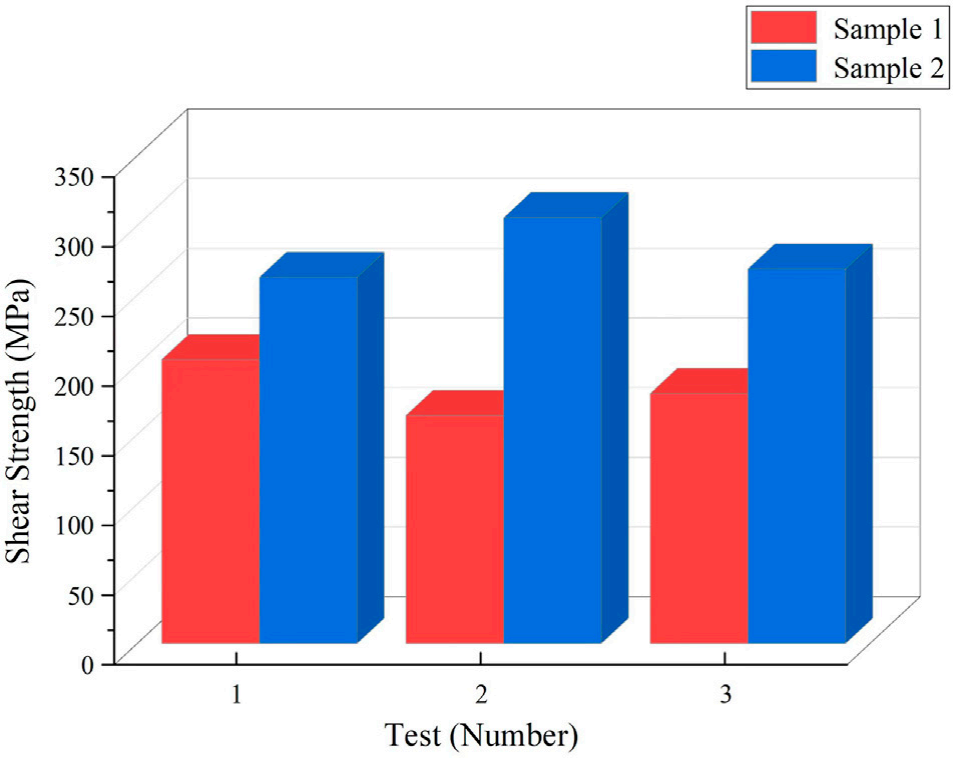
Shear strength of brazed joints with different brazing alloys.

Three shear tests were carried out separately for the two brazed joints, and the comparison of the mechanical properties of brazed joints is shown in Figure 7. Samples 1 and 2 represent alloy obtained by melting the circuit board and alloy obtained by melting pure metal, with average shear strengths of 182.21 MPa and 277.02 MPa, respectively. The brazing joint strength of alloy obtained by melting the circuit board is lower than that of pure metal melting alloy obtained by pure metal by 94.81 MPa. The fracture occurred at the brazed joints. According to the related.

**FIGURE 8:**
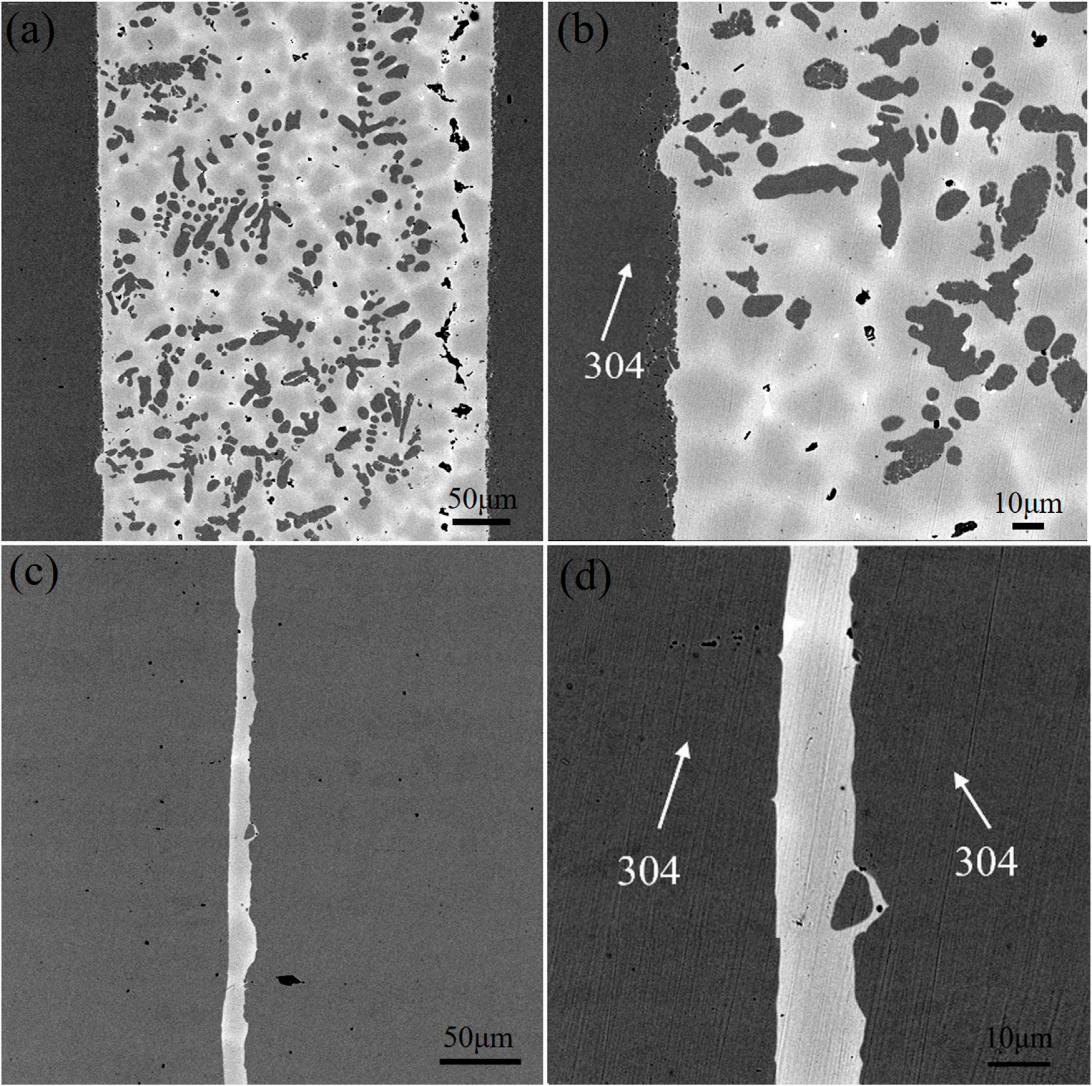
Microstructure of brazed joints with two brazing alloys **(A) (B)** brazed joint with alloy obtained by melting the circuit board and**(C) (D)** alloy obtained by pure metals.

Research (Wang et al., 2021), it is judged that the strengthening mechanism of alloy obtained by melting the circuit board is precipitation strengthening, while that of alloy sample No. 1 obtained by the pure metal is mainly solid solution strengthening. However, the alloy obtained by melting the circuit board appears segregated, resulting in the shear strength obviously inferior to the strength of the brazed joints of the No. 2 alloy. Figure 8 shows photos of the brazed joints microstructure about alloy obtained by melting the circuit board and alloy obtained by pure metal. From Figures 8A–B, it can be seen that the brazed joint seam of alloy obtained by melting the circuit board is densely.

**FIGURE 9:**
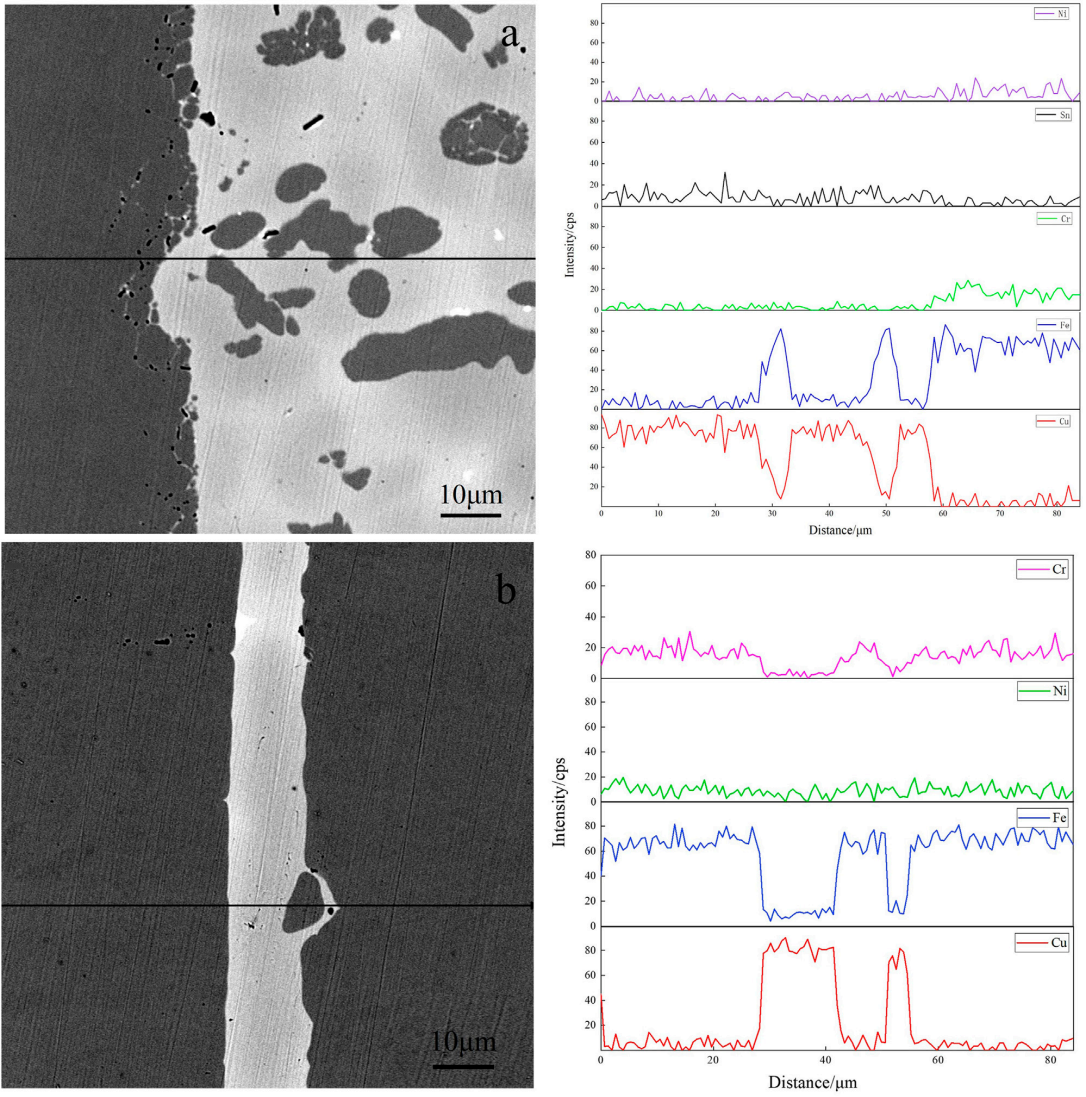
SEM image and EDS line scanning of elements with two brazed joints **(A)** alloy obtained by melting the circuit board and**(B)** alloy obtained by pure metals.

Organized, mainly with α-Fe dendrites and Cu matrix. Meanwhile, diffusion occurs at the interface between the brazing joint and 304 stainless steels, and the microstructure of base metal near the interface is partially dissolved and enters the brazing seam. In addition, the brazing seam is wider, ranging from 275 to 283 μm. As can be seen in Figures 8C,D, the brazing seam of alloy obtained by the pure metal is free of black α-Fe dendrites, and the brazing seam is mainly dominated by Cu matrix with the tiny microstructure of the brazing seam. The diffusion of the base metal can be observed on the high magnification image. Figure 9 shows the SEM images of the brazing seam and the line distributions of elements in two brazed joints by EDS. The line scan of Figure 9A from left to right through the areas of 304L stainless steel parent material, 304L-braze interface, and braze zone; Figure 9B from left to right for base metal of 304L stainless steel, 304L-brazing seam interface, brazing seam area, brazing seam-304L interface, the base metal of 304L. Figure 9B displays from left to the right base metal of 304L stainless steel, 304L- brazing seam interface, brazing seam area, brazed seam-304L interface, and 304L base metal. As can be seen from Figure 9, the width of the two brazing seam area and the corresponding intermetallic compounds are obviously different. At the interface between the base metal of 304L and alloy brazing filler metal obtained by melting the circuit board, the elemental content of Fe gradually decreases, and the elemental content of Cu gradually increases, which form a transition phase of diffusion distribution. Consequently, the microstructure and morphology of the interface region are closer to that of α-Fe dendrites without obvious demarcation. The interface between the brazing seam of alloy obtained by the pure metal and the base metal is similar to that of the brazing seam of alloy obtained by melting the circuit board, where the Cu and Fe elements diffuse into each other. Due to limited diffusion ability of Cu in Fe, the thickness of the brazing seam interface layer is only 13–20 μm with uneven thickness distribution.

## Conclusion

1) This study focuses on the microstructure and phase composition of the brazing alloys obtained by recycling and melting circuit boards. The alloys consist of α-Fe dendrites, (Cu, Sn) phase, Sn-rich phase, and copper matrix.
2) Compared to alloy obtained by the pure metal, the melting temperature range of alloy obtained by melting the circuit board expands, the melting temperature becomes higher, the shear strength is reduced, and the shear strength of the two multi-component brazing alloys is 182.21 MPa and 277.02 MPa, respectively. In addition, there are a large number of precipitated phases in the brazing alloy obtained by melting the circuit board, and the primary strengthening mechanism is determined as precipitation strengthening. Solid solution strengthening is the main strengthening mechanism of alloy obtained by the pure metal.
3) There are primarilyα-Fe dendrites and Cu matrix tissue with dense microstructure in the brazing seam of alloys obtained by melting circuit board. Diffusion occurs at the interface between the brazing seam and 304 stainless steels; The tissue of the base metal near the interface is partially dissolved and enters the brazing seam. In addition, the thickness in the brazing seam is wider, at 275–283 μm. The interface between the brazing seam of alloy obtained by melting the pure metal and the base metal is similar, where the Cu and Fe elements diffuse into each other; The thickness of the interface layer is only 13–20 μm with uneven thickness distribution due to the limited diffusion ability of Cu in Fe.

## Data Availability

The original contributions presented in the study are included in the article/Supplementary Material, further inquiries can be directed to the corresponding author.
